# Technologies and combination therapies for enhancing movement training for people with a disability

**DOI:** 10.1186/1743-0003-9-17

**Published:** 2012-03-30

**Authors:** David J Reinkensmeyer, Michael L Boninger

**Affiliations:** 1 Department of Mechanical & Aerospace Engineering, University of California, 4200 Engineering Gateway, Irvine, CA 92697-3875, USA; 2 Department of Anatomy and Neurobiology, University of California, 4200 Engineering Gateway, Irvine, CA 92697-3875, USA; 3 Department of Biomedical Engineering, University of California, 4200 Engineering Gateway, Irvine, CA 92697-3875, USA; 4 Department of Physical Medicine & Rehabilitation, University of Pittsburgh School of Medicine, 3471 5th Ave, Suite 201, Pittsburgh, PA 15260, USA; 5 VA Pittsburgh Healthcare System, 6425 Penn Avenue, Suite 400, Pittsburgh, PA 15206, USA

## Abstract

There has been a dramatic increase over the last decade in research on technologies for enhancing movement training and exercise for people with a disability. This paper reviews some of the recent developments in this area, using examples from a National Science Foundation initiated study of mobility research projects in Europe to illustrate important themes and key directions for future research. This paper also reviews several recent studies aimed at combining movement training with plasticity or regeneration therapies, again drawing in part from European research examples. Such combination therapies will likely involve complex interactions with motor training that must be understood in order to achieve the goal of eliminating severe motor impairment.

## Introduction

A key working hypothesis of rehabilitation science is that use-dependent plasticity perseveres through motor system injuries and diseases. This hypothesis drives intensive, ongoing efforts to optimize rehabilitation experiences for people with a movement disability, so as to best promote use-dependent plasticity. In the past twenty years, there has been an increasing recognition that technologies, including robotics, orthotics, wearable sensors, computer vision, computer gaming, electrical stimulation, virtual reality, machine learning, and computational modeling, can play an important role in these efforts [1-5]. In this section, we first review the rationale for developing this new technology for rehabilitation therapy, then, using examples from robot-assisted therapy, we briefly characterize the state of the field in meeting its promise. In the following sections we then review approaches to improve these technologies, drawing on examples from European research, followed by a brief discussion of attempts to combine these technologies with biologic therapeutics.

There are three primary motivations for developing new technology for rehabilitation therapy. First, improved technology has the potential to allow more therapy with less supervision, improving rehabilitation cost-benefit profiles. This objective can be expressed as developing technology that optimally promotes use-dependent plasticity while lowering the cost of therapy. Second, technology has the potential to more accurately quantify therapy, including patient characteristics that predict therapy success, the dose and content of therapy, and clinical outcomes. This quantification property of technology is important for improving the mechanistic understanding of rehabilitation science, clinician decision-making, and patient feedback and motivation. Third, technology has the potential to allow entirely new types of therapy. One example is the concept of providing continuous therapy with wearable devices. Rehabilitation therapists cannot be omnipresent, but smart, wearable technology almost can, providing therapy throughout the day as people participate in activities of daily living. The therapeutic effect produced by functional electrical stimulation (FES) foot drop stimulators, in which people who use the stimulators over an extended period of time exhibit improved walking ability even when they turn the stimulator off, is one example [6]. Another example of a promising new therapy that technology makes possible is manipulating limbs with robots in a way that precisely augments kinematic errors and thus enhances error-based learning [7].

Aiming to achieve these three goals, there has been a rapid increase in the development of therapeutic technology in the past 10 years, and a rapid growth in commercial products for rehabilitation training [1-5]. However, results with this technology are mixed so far, and when and in what form this technology will deliver the desired improved outcomes for rehabilitation is unclear. We illustrate the state of the field with three recent studies of robot-assisted movement training after chronic stroke.

Most clinical trials of robot-assisted movement training have used robots to physically assist the limbs of patients as they attempt desired movements and/or play computer-guided activities and games. Thus, the robots tested so far have typically implemented a technique from physical rehabilitation called "active assist therapy", in which the patient actively tries to achieve a movement as the therapist manually assists in the movement. Besides allowing a patient to perform movements not possible without assistance, it is thought that active assist therapy may generate new patterns of sensory input that may influence brain plasticity.

The first robot therapy study that illustrates the state of the field is the recent multi-center randomized controlled trial of robot-assisted therapy sponsored by the Department of Veterans Affairs [8]. In this study, 127 people with chronic stroke were randomized to receive either 1) robot-assisted upper extremity training with three modules of the MIT-Manus robot; 2) upper extremity exercise with a rehabilitation therapist that was matched in number of movements to the MIT-Manus therapy and therefore was characterized as "intense", or 3) usual care. Robot-assisted therapy was significantly more effective than usual care, but the benefits were small--about two additional points on the upper extremity Fugl-Meyer scale, which ranges from 0 for complete paralysis to 66 for normal movement ability [9]. Robot-assisted therapy was about as effective as the intense, therapist-delivered therapy, although as follow-up time progressed the patients who received the robot-assisted therapy exhibited a trend of more motor gains. The cost of delivery of the robotic and therapist-delivered therapies was similar, in large part because of the relatively high costs of the robots used in the study. However, the amount of therapy delivered was much greater than what would normally occur in an inpatient or outpatient rehabilitation setting. Thus, if the costs of robotics decrease it may be possible to deliver this therapy-intensive care, while delivering this type of care in the absence of robotics will likely never occur. Detailed analysis of the sensor-based data from this study is forthcoming, but, previous analysis of data obtained from similar MIT-Manus studies has been used to suggest that recovery is fundamentally characterized by a progressive blending of sub-movements [10].

In another study, 48 people with chronic stroke who were ambulatory at study start were randomized to train walking using a treadmill and the Lokomat gait robot, or a treadmill with manual assistance from a physical therapist [11]. For the Lokomat training, the participants did not receive biofeedback about their contribution to the walking motion. Training with either approach produced modest but measurable benefits in walking speed; training with the Lokomat and without biofeedback was about half as effective as the therapist-delivered training in terms of improvement of gait speed. It has been hypothesized that the relatively rigid robot assistance without biofeedback, as provided in this study, may have been less effective because it caused patient slacking [11]; analysis of oxygen consumption during such training [12], as well as computational modeling of the evolution of interaction forces during robot-assisted training [13], quantitatively support this idea. Another possibility is that the rigid assistance reduced variability needed for learning [14]. Analysis of training data from the study itself showed that Lokomat training as implemented was indeed less variable [15]; analysis of fixed robotic gait training in rodents with SCI suggests that rigid assistance that does not allow kinematic variability tends to disrupt muscle activity [16].

In a third study, 28 people with moderate to severe arm impairment due to a chronic stroke were randomized to participate in training with a passive arm exoskeleton called T-WREX or in standard table-top exercises with no technology [17]. T-WREX simulated functional activities using computer games and an anti-gravity arm support orthosis which also incorporated a grip sensor that allowed patients with even trace amounts of grasp to participate in simple grasp-and release actions to control the games. Both groups only required about 3 minutes of therapist contact following a week of training, as measured by stopwatch. Both groups improved their arm movement significantly by about 2-3 FM points; at 6 month follow-up the T-WREX group had significantly better scores, although the difference was small (2 FM points). When given a chance to try the other therapy then asked to subjectively compare the two approaches, the participants expressed a strong preference for the technology-based approach, finding it more motivating in part because the arm weight support improved their perception of self-efficacy [18].

One can extrapolate broader themes that characterize the general state of technology for rehabilitation therapy from these three illustrative studies. First, considering the goal of improving cost-benefit profiles, one can observe that technology-assisted exercise produced significant benefits in all three of the studies reviewed above, and that it is sometimes possible to use technology with only small amounts of therapist supervisory time, as directly measured in the T-WREX study. These observations support the premise that, indeed, some aspects of rehabilitation therapy do not require the immediate presence of a rehabilitation therapist to be effectively implemented with technology. However, the cost of the technology used for this substitution may still limit cost-benefit profiles, as was found, for example, in the cost analysis of the MIT-Manus study, indicating a need for lower cost technologies.

Second, considering the goal of quantification, while it is true that there is potentially a computer record of every force or movement the participants made during training in these studies, we are just beginning to understand how to use this data to predict responders, guide therapy, or define mechanisms of recovery. For example, as mentioned above, data from MIT-Manus has been used to identify a role for sub-movement blending in movement therapy, and data from the Lokomat and rodent robotic devices was used to analyze the role of kinematic variability in training. Thus, the field is just beginning to develop ways to use data from sensors incorporated into rehabilitation technology to provide insight into use-dependent plasticity.

Third, considering the goal of innovating to produce new forms of therapy that are more effective, it is apparent that some innovations in technology-based therapy are as effective as therapists for particular forms of training, few or none are more effective, and many are less effective. The reasons are complex and poorly understood at present, but a key limitation that must be overcome is improving the hardware and control design of these devices to increase efficacy. Understanding the reasons particular implementations decrement learning, while other implementations increment learning, is important. At present, one might say that the only innovation that new technologies routinely make available, besides semi-automation of training, is that of a more motivating context for rehabilitation training, by virtue of helping patients achieve movements or simulated activities that they normally could not, and by providing a computer gaming context with quantitative feedback to motivate practice.

## Promising directions for technology-enhanced therapy

Given this current status, how can physical therapeutic technology be improved? Several key themes emerged during our panel's visit to Europe.

### Designing technology for early application after injury

The healthcare environment in some countries in Europe has made it easier to test therapeutic technologies earlier in rehabilitation, as patients are permitted to stay in sub-acute rehabilitation facilities much longer than in the United States. Landmark studies by the group of Dr. Stefan Hesse at Charite Hospital, Berlin, found large improvements in motor function of both the upper [19] and lower [20] extremities when robot-assisted training was provided early after stroke. This work supports the concept that the motor system exhibits a temporal window early after injury in which plasticity is relatively enhanced. The existence of this window is an important consideration for technological design because the subacute rehabilitation environment imposes design constraints on the technology to be used in the environment, since patients tend to be more impaired and stay in bed more in subacute rehabilitation. The robotic therapy group of the University of Padua has therefore, for example, developed a device that can specifically be used at bedside to provide early mobilization of the flaccid arm [21]. This group again found larger changes in arm function due to robot-assisted therapy than have typically been reported with therapy delivered in the chronic phase post-stroke [22]. However, despite these promising results, there are still relatively few studies applying therapeutic technology soon after injury. This is likely also due to complications associated with such studies, including patients having severe, concurrent medical issues, the confounding nature of natural recovery, and the relatively high intensity of therapy already provided in this stage, which may not leave time for additional test interventions. In summary, a key direction for rehabilitation technology is to develop and test devices specifically for early training after neurologic injury.

### Designing lower cost devices

**Figure 1:**
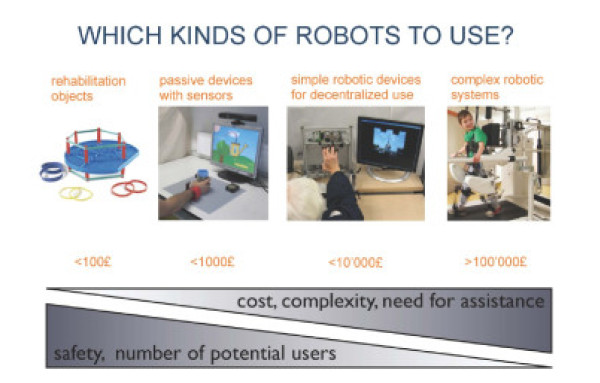
The spectrum of complexity in rehabilitation theraphy technology, ranging from simple rehabilitation devices (*left*) to complex robotic systems (*right*) (courtesy of Dr. Etienne Burdet, Imperial College, London).

Another trend in the field is to develop lower-cost devices. Professor Etienne Burdet of Imperial College has observed that there is a spectrum of complexity in technology for rehabilitation therapy, starting with simple rehabilitation objects already commonly used in rehabilitation therapy, to passive devices with sensors, to simple robotic devices for decentralized use, to complex robotic systems (Figure 1). Moving along this complexity spectrum increases cost and the need for assistance from humans to use the technology, while decreasing safety and the number of potential users. Dr. Burdet has therefore focused his work in the middle of the spectrum, on passive devices and simple robots that could potentially be accessed by more people than the existing, more complex commercial products (e.g., [23]).

The work of Dr. Hesse is also of note again in this regard, as the robotic devices that produced the excellent clinical results mentioned above, which are now sold by a start-up company RehaStim, are relatively simple, low degree-of-freedom robotic devices for both the upper and lower extremities. Another world leader in commercializing therapeutic technology, Hocoma A.G., recently released a simplified arm therapy device, ArmeoBoom, based on the work at U. Twente and Roessingh Rehabilitation Center [24]. Major companies, such as Phillips [25] and Intel [26] have developed relatively low cost sensor-based systems for home-based therapy. An interesting possibility is to use cell phone platforms to drive therapy, an approach pursued by the Tril group in Ireland http://www.trilcentre.org/media/news/tril-at-eric-conference.html, the Department of Electronics, Computer Sciences and Systems, at the Università Di Bologna http://www3.deis.unibo.it/en, and others. Software on cell phones combined with movement sensors could monitor exercise performance and compliance, test a user's physical status, and provide encouragement, motivation, and feedback. Gaming consoles, such as the Nintendo Wii, the Microsoft Kinect, and the Sony Playstation Move, although not technically for rehabilitation, could be adapted for training and assessment, and custom computer games can be developed as well [5]. Despite this work, there has not yet been a breakthrough: there are still no devices or software specific to rehabilitation that people with a mobility impairment routinely use at home to engage in rehabilitation therapy. Issues of safety, remote progress assessment, data mining, and remote interaction between therapist and patient will have to be solved. For more discussion of these issues, the reader is referred to the companion paper by Patel et al. on wearable sensors for rehabilitation in this issue.

### Developing technology with more degrees of freedom

**Figure 2:**
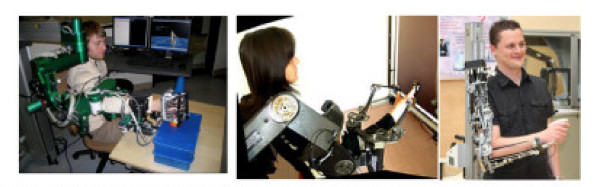
**Example upper extremity exoskeletons with at least four degrees of freedom, including ARMIn from ETH Zurich (*left*, 27), the L-Exos from Scuola Superiore Sant'Anna, Pisa, Italy (*middle*, 28), and the Able Exoskeleton from CEA/ISIR/Haption in France (*right*, 29)**.

At the same time that many groups are working to develop simpler technology, other groups worldwide are increasing the mechanical sophistication of the technology to be used in rehabilitation. The rationale for this work is that training more naturalistic movements may improve functional outcomes. For the upper extremity, groups at ETH in Zurich [27], Scuola Superiore Sant'Anna, Pisa, Italy [28], and CEA/ISIR in Paris [29] are examples of work to develop exoskeletons that allow naturalistic movement of the arm by accommodating at least four degrees of freedom of shoulder and elbow movement (Figure 2). The ETH exoskeleton, ArmIn, is now being commercialized by Hocoma. The exoskeleton at ISIR, commercialized by Haption, is particularly lightweight and comfortable, owing in part to patented actuators first manufactured at the CEA and carefully designed passive degrees of freedom that accommodate joint rotation center mismatches [30], a strategy also proposed by a group in the Netherlands [31].

For the lower extremity, work is underway at ETH Zurich, University of Pisa, and Hocoma to add pelvic and ankle degrees of freedom to the Lokomat, and concepts from the simple gait-trainer developed by Hesse have been expanded in collaboration with Fraunhofer IPK to produce the Haptic Walker Gait Trainer 2 [32,33]. This device consists of two six degrees-of-freedom foot plates that can support the weight of the patient and be programmed to simulate different step characteristics, including stair walking. Another device developed at Fraunhofer IPK that allows naturalistic movement for training balance and posture over a treadmill is the StringMan [34]. It consists of eight force-controlled pulleys attached to a harness worn by the patient. It can be programmed to provide a virtual envelope in six degrees of freedom for the trunk and pelvis to support the patient during training. Two other examples of sophisticated gait trainers being developed in Europe are the LOPES gait trainer, developed at the University of Twente, which uses cable-driven, series elastic actuators to provide compliant assistance to naturalistic gait movement [35], and the Walktrainer robot at EPFL, which moves along with the patient as it assists in leg movements [36]. Clinical testing with these devices is ongoing; this testing will help determine whether training more naturalistic movements will indeed improve functional outcomes enough to justify the added cost and complexity of these devices.

### Wearing the therapeutic technology

Another major trend in technology for rehabilitation therapy is to make the technology wearable. Wearable sensor systems for therapy are reviewed in a companion article in this special issue. The rationale for developing actuated, wearable orthotic systems is, again, to make training more naturalistic, and ultimately to free training from the confines of the rehabilitation clinic. This freeing of training will break down the current distinction between assistive and therapeutic technology; people will use therapeutic technology to assist them in activities of daily living, undergoing therapy at the same time as achieving desired tasks. Such technology will be designed to continuously adapt to the user, to appropriately challenge them and progress training. This dual-purposing of therapeutic technology will increase the dosage of therapy beyond levels possible in the clinic only, and increase the likelihood that what is learned during training will be useful in the real world, since training will be in the real world.

Examples of wearable systems developed in Europe include the ActiGait system, sold by Neurodan/Otto Bock. ActiGait is a multi-channel, implantable functional electrical stimulation system for foot drop based on research at the University of Aalborg [37]. Another example is the work of Dr. Jose Pons in Madrid. Dr. Pons has developed an innovative knee-ankle-foot orthosis that can assist people with leg weakness in achieving normal joint kinematics during walking [38]. The contribution of the joints to different phases of the gait cycle is approximated using spring-like, force-length curves, and actuators for each joint are constructed of compression and tension springs. The actuators use solenoids or an ankle-driven Bowden cable to switch between springs to reproduce the desired spring characteristics during each phase of the gait cycle. The system has been tested with users who have poliomyelitis and shown to improve the gait pattern [38,39], and is being investigated for commercialization by Össur.

### Improving control and feedback

There are intensive efforts worldwide to improve the control algorithms and patient feedback algorithms for therapeutic technology. One concept is to make robotic therapy devices patient cooperative, as proposed by Professor Robert Riener at ETH Zurich [40], a strategy which can improve active participation of the patient [41]. Path control provides a virtual 'tunnel' in which the patient can modify his or her steeping pattern [42]. Guiding forces are applied when the person begins to deviate beyond the 'tunnel' boundaries. This group is also developing control algorithms for canceling the inherent inertia of exoskeletons, so that the patient feels less of the robot [43]. The group at University of Genoa has designed innovative algorithms for adaptively reducing robot assistance during training [44], providing progressive challenge. Recognizing that robotic training can be passive and boring, others are developing virtual reality systems to engage patients, using visual and auditory inputs [45]. Physiological monitoring of exercise markers such as cardiovascular response is also being explored as a means to adapt training [46].

Another approach is to combine brain computer interface technology or functional electrical stimulation technology with robotic therapy. An example of the use of BCI technology for therapy comes from Dr. Pons, who is also the Project Coordinator for The BETTER project (Brain-Neural Computer Interaction for Evaluation and Testing of Physical Therapies in Stroke Rehabilitation of Gait Disorders). This project is focused on improving physical rehabilitation therapies by combining brain computer interfaces (BCI) with wearable exoskeletons and robotic gait trainers, such as the Lokomat. The BCI being used is an EEG-based BCI. The goal is to encourage brain plasticity by programming the robot to exert physical stimulation at the periphery as a function of the neural activation patterns at the brain. One possible benefit of this approach is to intelligently promote active participation of patients during therapy. Training of the BCI parameters may still be possible even for a person who is completely paralyzed by tapping into the mirror neuron system [47]. Mirror neurons fire when an action is observed and robotic exoskeletons may be able to move a patient's arms while a BCI records signals that can be used later for control. Other recent work combining BCI with robotics or FES for therapy includes [48,49]. Several other groups in Europe are combing electrical stimulation with robotics [36,50,51], a strategy that ensures that muscles stay active during repetitive, guided training.

### Modeling the mechanisms of therapy using computational neuroscience

Ideally, therapeutic technology would be designed based on experimentally verified mathematical models of how limb use drives plasticity, in the same way that new materials can be designed based on a fundamental knowledge of chemistry and solid mechanics. Such models do not exist yet; the field of "neurocomputational rehabilitation" is nascent [52-56]. One key development with European contribution in this field is a computational model that explains how the motor system coordinates muscles to achieve impedance control, internal model formation and effort optimization when interacting with a dynamic environment [57]. In the model, the motor system modifies motor commands to the muscles based on kinematic error sensed locally at individual muscles, using a simple, sunken, asymmetric, "V" function. This model explains a wide range of experiments from the motor adaptation literature in which humans interacted with dynamic robotic environments [57,58]. This model can likely provide a "low level" basis for helping understand patient response to physically-interactive rehabilitation therapy, as well as orthotic and prosthetic devices. However, there is still clearly a great need for "high level" models of motor plasticity and learning that are built on top of this low level. The Berlin research institute Fraunhofer IPK has identified the development of a comprehensive model of motor control and motor learning, in which the human is seen as a biocybernetic system, as a grand challenge for the field of mobility technology, and has worked toward the development of a major center involving several groups across Germany focused on this problem. They see a need for an integrative model of orthopedic, muscle, and neural plasticity that can be used as a basis from which to design innovative mobility technology.

### Combination therapies

Improvements in motor performance following rehabilitation therapy, with or without technology, are often modest. While refinements in rehabilitation technology will likely improve clinical outcomes by making therapy more available, more motivating, and perhaps more targeted and effective, it is also likely that recovery will ultimately be limited unless the damaged or diseased biological systems responsible for the motor impairment are restored. We define combination therapies as rehabilitation strategies that combine drug, molecule, or cell-based therapeutics with technology for movement training. There is already evidence that loading, training, and exercise will be important for facilitating biologic therapeutics, including regeneration of skeletal and cardiac muscle, bone, and neural systems. For example, physical therapeutics appear to establish a more permissive microenvironment and help direct cell fate for regeneration [59]. In the central nervous system it is likely that neural cues will be needed for regenerative therapeutics to cause effective cell differentiation and generation of needed neural pathways. A key question, however, is whether any reasonable exercise that is delivered in combination with biologic therapies will be effective, or whether there will be subtlety in the way that exercise synergizes with biologic therapeutics, and therefore a need for optimization. Recent studies examining biological therapeutics in neural injury suggest that combination therapies involve complex interactions with motor training that must be understood in order to achieve the goal of eliminating severe motor impairment [60,61].

A key example comes from Dr. James Fawcett's group at Cambridge University, which has been working with chondroitinase ABC, a bacterial enzyme that digests molecules that help form cartilage-like barriers to axonal growth. Using a rat model of a spinal cord injury that disrupted the corticospinal tract, they found that delivering chondroitinase to the injury site without training the rat to use its impaired paw was ineffective, where the outcome measure was the number of sugar pellets the animal retrieved from a stair-cased well [62]. They studied this task because it has been shown previously to require a corticospinal tract [63], which was the tract targeted with the lesion in their study. They then found that delivering rehabilitation exercise specific to paw reach and retrieval for one hour per day, in the form of practice at retrieving seeds embedded in a plastic floor grid, led to an impressive recovery of skilled paw function, but only when coupled with chondroitinase treatment. Interestingly, delivering generalized forelimb rehabilitation for one hour per day in the form of an enriched environment (or "fun cage" with ladders, ropes, and tunnels), extinguished the rat's ability to perform the pellet retrieval task, whether or not they received chondroitinase.

**Figure 3:**
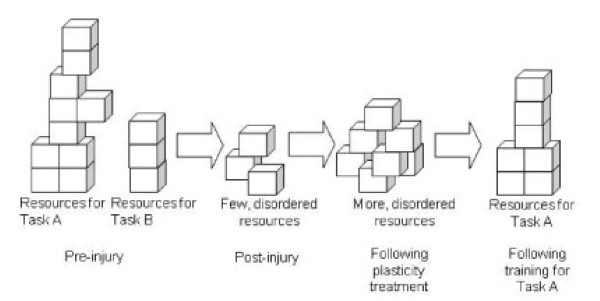
**Conceptual diagram of competition of task-related motor circuits for new neural resources made available with a plasticity treatment**. Neural resources, such as synaptic connections, are represented by blocks. Pre-injury, there are ample resources to support motor control of multiple tasks. Following a neural injury, there are fewer resources and they are disordered. Following a plasticity treatment, there are more resources, but they are still disordered. Training on motor Task A results in ordering of blocks for that task, but leaves no blocks for building a controller for Task B.

One interpretation of these results is as follows (Figure 3). The plasticity treatment chondroitinase induced axonal sprouting; rehabilitation exercise pruned and connected the sprouts. Thus the new neural resource made available during a window of time by chondroitinase was wasted without rehabilitation exercise. Practicing a target motor skill (i.e., skilled paw retrieval) appeared to recruit the newly available neural resources to serve and improve the skill. Practicing other motor skills (as the rats did in the fun cage), appeared to negatively affect the learning of skilled paw use. Thus, there may exist a neural competition for the new neural resources induced by plasticity treatment. The type of movement practice experienced may drive the competition.

Other work has found similar evidence of competition in training. For example, rats with lesions of the corticospinal tract who were trained in skilled reaching improved in reaching ability, but made more errors in a horizontal ladder test [64,65]. The presence of this phenomenon depended on which anatomical component of the corticospinal tract was lesioned. Another study examined the individual and combined effects of locomotor training and treatment with the Anti-Nogo-A antibody, which helps prevent inhibition of neurite outgrowth following spinal cord injury in rats [66]. Both therapies improved locomotor function, but in different ways, as detected by kinematic analysis of hindlimb movement. Combined treatment actually decreased functional performance on a ladder climbing task, suggesting that the mechanisms underlying the treatments were again competitive. It was noted that this interference may depend on the relatively timing of delivery of the two therapies [66]. Motor training combined with another axonal growth promoter after a focal cortical infarct in rats produced primarily temporal benefits: recovery of grip function was better early [67], supporting the concept that temporal dynamics will be important in combination therapies. In another recent study, genetic deletion that reduced myelin-mediated inhibition of neural plasticity in mice combined with a novel form of technology-enabled exercise training that simultaneously challenged balance, grasping, and locomotion after partial lateral hemisection exhibited differing effects, with genotype providing improved performance on more generalized behaviors, and training a task-specific benefit, with no observed additive effect [68].

Another important example is provided by the group of Prof. Gregoire Courtine of the Experimental Neurorehabilitation Lab at the University of Zurich. This group uses animal models of SCI to help inform their clinical use of robotic devices, and is investigating use of electrical stimulation of the spinal cord for motor function combined with neuropharmacological interventions, in both murine and non-human primate models. One recent study from this group with a remarkably comprehensive quantitative analysis coupled robotic locomotor training, pharmacological intervention, and epidural electrical stimulation in rats with a complete SCI [50]. The combination of approaches produced additive effects that allowed the injured rat to walk nearly normally. These authors therefore argued that the diffusely distributed and heterogeneous character of neuromotor control systems demand multiple complementary approaches.

This work has clear implications for engineering approaches to rehabilitation exercise. In the words of Dr. Fawcett and Dr. Armin Curt, "the plastic CNS may be very vulnerable to poorly planned rehabilitation" [61]. Physical therapeutic technologies may help provide control over which functions are reprogrammed, and therefore may help to maximize synergism and minimize competition. Physical therapeutic technologies may also be useful for assaying the amount of and type of plasticity made possible by a treatment, so that rationale decisions can be made about what motor skills to train. Finally, there is a critical need for neuro-computational models that can be used to understand the competitive and synergistic interactions between different types of movement practice and biologic therapies.

## Conclusions

There is an explosion in new rehabilitation technologies; however, the field is in its infancy. Beyond the fact that these technologies can in some case make rehabilitation exercise more engaging and less labor intensive, the gains delivered are still unclear. Fundamental scientific insight is needed into the learning and plasticity mechanisms that these technologies seek to stimulate; the current lack of insight makes device design somewhat haphazard. Nevertheless, promising areas of development include developing technology for delivering therapy both earlier in clinics, and later at home; investigating the relative roles of both simpler and more complex technology in promoting plasticity, thereby testing the premise that training with more naturalistic movements will better promote functional recovery; making devices wearable to extend the reach of training to the lived-in environment; improving feedback and implementing learning-based control to make training more engaging and challenging; and coordinating multiple therapeutic modalities, including robotics, FES, and BCI's to enhance the effect of training. A new field of neuro-computational rehabilitation appears to be developing, in which computational models will be used to simulate and understand use-dependent plasticity in rehabilitation therapy. Regenerative therapies may enable levels of recovery far beyond those possible with rehabilitation exercise alone, but these therapies cannot progress independently of rehabilitation exercise.

Thus, the challenge of developing technologies that significantly improve on rehabilitation outcomes compared to conventional rehabilitation remains to be met. The quantification power associated with sensors incorporated into therapeutic technologies, coupled with the nascent field of neuro-computational rehabilitation will help resolve this gap. We expect there to be a "science of combination therapies" that seeks to understand the complex interactions between training, plasticity, and regeneration [59,60]. The most effective physical therapeutic technologies of the future will likely be based on this science.

## Competing interests

David Reinkensmeyer has a financial interest in Hocoma, A.G., a company that makes robotic therapy devices. The terms of this arrangement have been reviewed and approved by the University of California, Irvine, in accordance with its conflict of interest policies.
